# Accuracy of low-cost alternative facial scanners: a prospective cohort study

**DOI:** 10.1007/s10006-022-01050-5

**Published:** 2022-03-05

**Authors:** Alexander K. Bartella, Josefine Laser, Mohammad Kamal, Matthias Krause, Michael Neuhaus, Niels C. Pausch, Anna K. Sander, Bernd Lethaus, Rüdiger Zimmerer

**Affiliations:** 1 Department of Oral and Maxillofacial Surgery, Leipzig University, Liebigstraße 12, 04103 Leipzig, Germany; 2 Department of Surgical Sciences, Faculty of Dentistry, Kuwait University, Safat, Kuwait

**Keywords:** Face scan, Three-dimensional face scan, Photogrammetry, Smart device, iPhone

## Abstract

**Introduction:**

Three-dimensional facial scans have recently begun to play an increasingly important role in the peri-therapeutic management of oral and maxillofacial and head and neck surgery cases. Face scan images can be generated by optical facial scanners utilizing line-laser, stereophotography, or structured light modalities, as well as from volumetric data: for example, from cone beam computed tomography (CBCT). This study aimed to evaluate whether two low-cost procedures for the creation of three-dimensional face scan images were capable of producing sufficiently accurate data sets for clinical analysis.

**Materials and methods:**

Fifty healthy volunteers were included in the study. Two test objects with defined dimensions (Lego bricks) were attached to the forehead and the left cheek of each volunteer. Facial anthropometric values (i.e., the distances between the medial canthi, the lateral canthi, the nasal alae, and the angles of the mouth) were first measured manually. Subsequently, face scans were performed with a smart device and manual photogrammetry and the values obtained were compared with the manually measured data sets.

**Results:**

The anthropometric distances deviated, on average, 2.17 mm from the manual measurements (smart device scanning deviation 3.01 mm, photogrammetry deviation 1.34 mm), with seven out of eight deviations being statistically significant. For the Lego brick, from a total of 32 angles, 19 values demonstrated a significant difference from the original 90° angles. The average deviation was 6.5° (smart device scanning deviation 10.1°, photogrammetry deviation 2.8°).

**Conclusion:**

Manual photogrammetry demonstrated greater accuracy when creating three-dimensional face scan images; however, smart devices are more user-friendly. Dental professionals should monitor camera and smart device technical improvements carefully when choosing and adequate technique for 3D scanning.

## Introduction

Obtaining three-dimensional (3D) surface images of the face and head area is increasingly desirable in various specialties. Generated images are used for preoperative planning and treatment simulation, patient education, postoperative evaluation, research, and in the fabrication of computer aided planning/computer aided design (CAD/CAM) products such as customized facial masks and surgical guides. Despite rapid advances in scanning technology, only a small number of professional camera systems which are dedicated solely to face and head scanning for medical purposes are currently available. Several variations of these facial scanning devices are available, either as mobile scanners such as Artec Eva (Artec 3D, Luxembourg), M4D Scan (Rodin4D, Mérignac, France), and Vectra H1 (Canfield Scientific Inc., Parsippany, USA), or as stationary scanning devices such as FaceScan3D (3D-Shape GmbH, Erlangen, Germany), Vectra M3/XT (Canfield Scientific Inc., Parsippany, USA), and 3dMD Face System (3dMD LLC, Atlanta, USA). Additionally, some cone beam CTs (CBCTs) provide a camera for face scans, which is particularly useful, as both soft and hard tissue can be considered in preoperative planning.

Furthermore, facial scanners are distinguished according to the optical techniques employed in generation of the 3D data set. Stereophotogrammetry involves taking several photos with multiple cameras from different angles, which are then merged to form a three-dimensional model [1]. An additional method called “triangulation” requires a similar technical device but uses structured light to capture the images. The capturing technology is based on the projection of parallel stripes on the patient’s face to create a pattern, which is then captured by cameras in order to reconstruct a 3D model based on mathematical algorithms which consider the distortion of the light pattern. These different capturing techniques have various advantages and have been extensively evaluated in the literature. Current face scan techniques are able to produce 3D face scan images with an accuracy of 0.32 to 0.89 mm [2–6].

Nevertheless, all available face scanners bear certain inherent disadvantages. First of all, the cost of a professional face scanner currently ranges from EUR 9000 to 95,000, thereby making them relatively expensive even for established clinical centers and research institutions. Furthermore, some devices require frequent recalibration and professional handling. Another drawback of stationary scanning devices is the need for a large space suitable for their permanent installation [7]. The abovementioned drawbacks necessitate the discovery of a cheap yet accurate alternative technique to obtain suitable face scan images. Smart devices are one promising option. In 2017, Apple Inc. introduced the TrueDepth camera in the iPhone X, which allows identification using face recognition. According to Apple Inc., 30,000 invisible points are projected and analyzed before an infrared image of the face is recorded. In addition to their wide availability, a great advantage of smart devices is their lower acquisition costs compared with most existing professional face scan systems.

Another promising technique that can be used to create 3D facial models is manual photogrammetry, which is based on the digital fusion of a series of single overlapping two-dimensional photos of the face from one or several cameras in order to create a 3D object [8]. A special photogrammetric method is the “structure from motion” (SfM) photogrammetric range imaging technique. In order to find matches between the images, certain points are tracked from one image to the next. In the SfM imaging technique, this process is fully automatic, whereas with conventional photogrammetry, manual determination of matching points is necessary for the calculation of a 3D surface.

Reviewing all of the current imaging modalities ultimately leads to the question of whether these rapidly evolving low-cost optical technologies can be used to create 3D models of the face while meeting the high demands of medical contexts. Therefore, the aim of the present study was to evaluate the accuracy of 3D facial images obtained using mobile smart devices and conventional photogrammetry-based optical scanning devices, and to compare the ability of these methods to create accurate 3D surface scans of the human face.

## Materials and methods

### Participants

Fifty healthy volunteers were included in this prospective study. The gender distribution was 22 to 28 (m:f). The mean age of the study population was 44.4 ± 16.4 years, ranging from 18 to 83 years. Exclusion criteria for the recruitment of participants were visible facial deformities and excessive facial hairiness (beards). The study protocol was approved by the institution’s ethical committee (083/20-ek) and was performed in accordance with the principles of the Declaration of Helsinki. Written informed consent was obtained from all volunteers prior to the study.

### Face scan and data acquisition

Data acquisition and elaboration were performed by the authors (J.L. and A.B.). Both authors contributed to the corresponding work to ensure that the results were of appropriate quality.

**Fig. 1:**
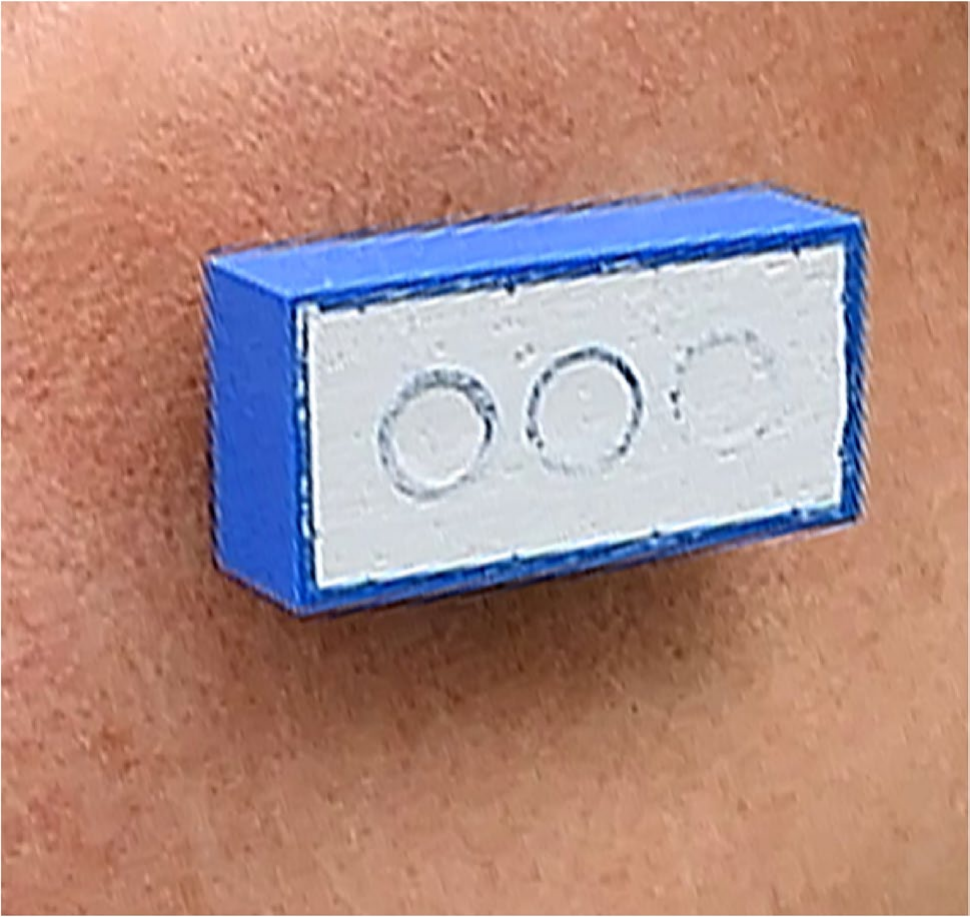
The distances and angles of a gaming brick are always the same and are therefore especially useful when testing the accuracy of cameras

**Fig. 2:**
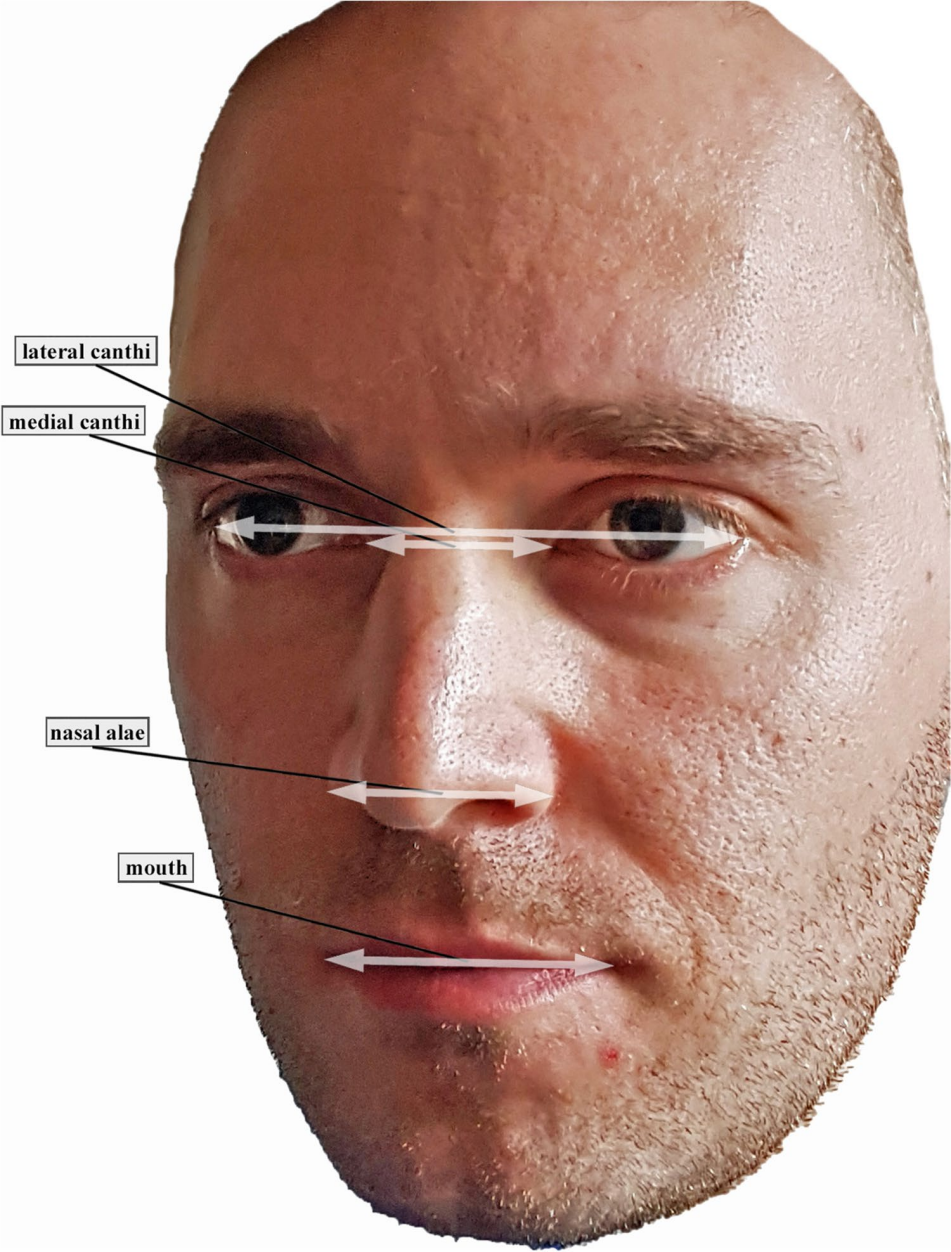
The anthropometric distances that were measured virtually

For better objectivity and comparability with the existing literature, anthropometric values and two geometric bricks (Fig. 1) were scanned and measured. Adequate exposure was ensured during recording and image capturing, and the volunteers were instructed to keep a natural head position, maintain a neutral facial expression, and not move their head. Reproducible anthropometric points and distances on the faces were measured manually. Distances between the medial canthi, the lateral canthi, the nasal alae, and the angles of the mouth were measured (Fig. 2). Distances between the anthropometric points on the volunteer’s face were measured manually with a circle. The data were documented and set as standards since they represented the real values.

**Fig. 3:**
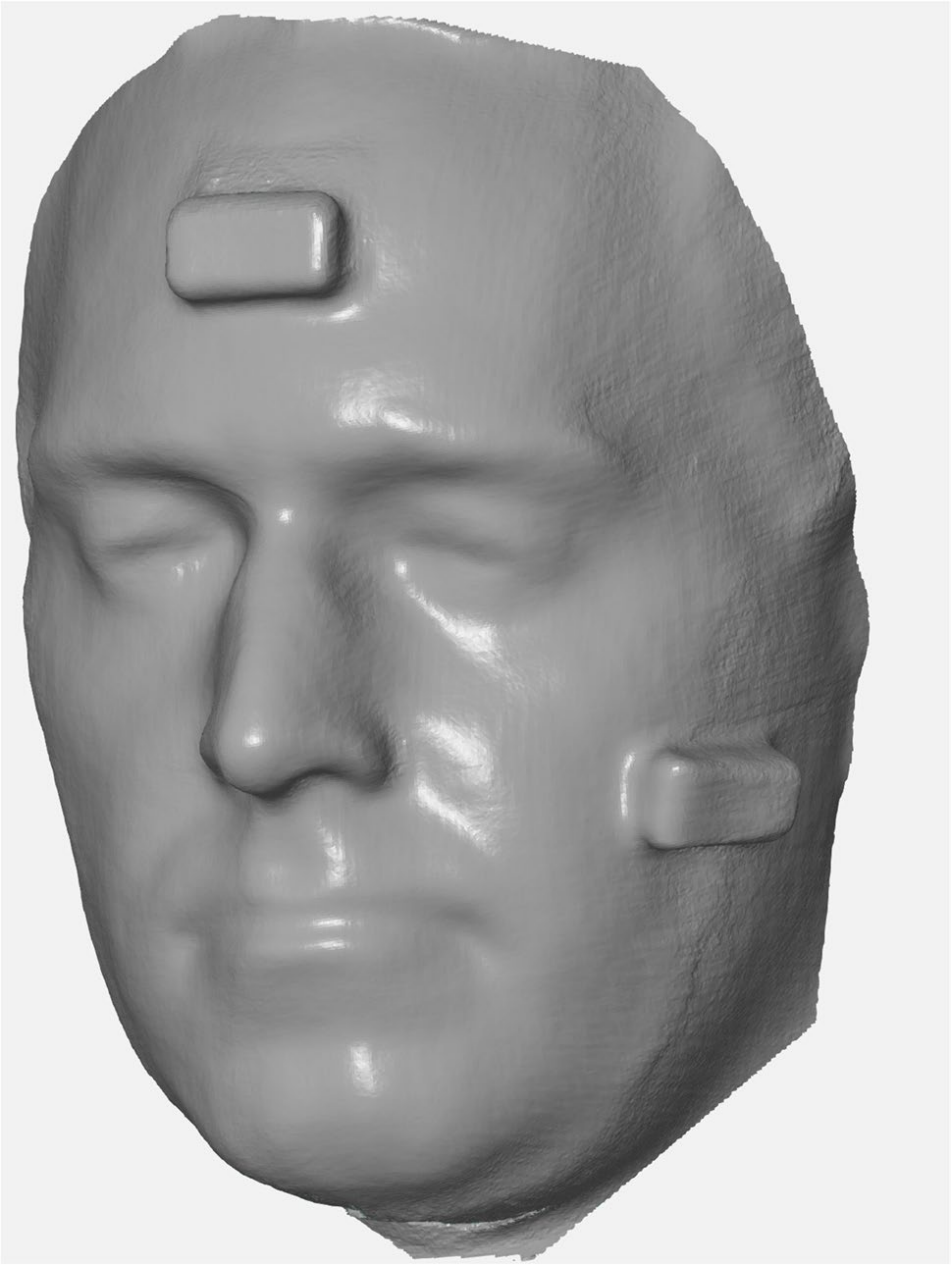
Face scan created by a smart device. The appearance of the face is smoother. However, the bricks are also affected by the smoothening and appear to be less accurate

**Fig. 4:**
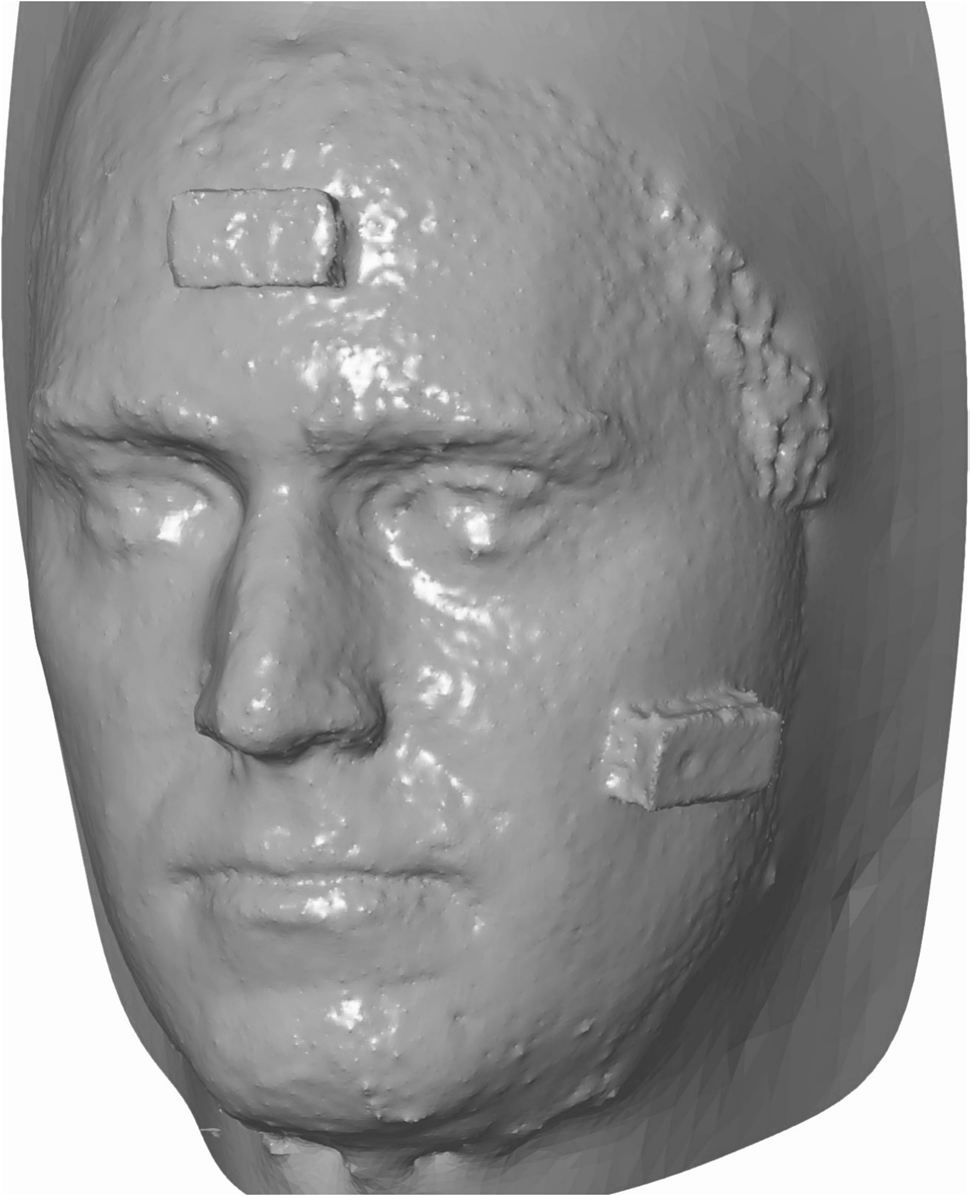
Face scan created using manual photogrammetry. For this particular scan, the fusion of a total of 151.18 ± 30.26 pictures was necessary. The uneven edge surface of the face is notable

Subsequently, two gaming blocks (the LEGO Group, Billund, Denmark) with uniform dimensions of 31.8 × 15.8 mm and angles of 90 degrees were used as markers. To achieve an even surface, the blocks were filled with plaster (Fig. 1). The two test items were then attached to the participant’s face. One was placed in the middle of the forehead and the other on the left cheek (Figs. 3 and 4). An iPad Pro (3rd generation, Apple Inc., Cupertino, USA) running the Heges app (version 1.2.4, developer: Marek Šimoník) served as the smart device for capturing the images. The cost of a new iPad Pro was approximately EUR 1099 and the software cost EUR 2.99. This software uses the front camera of a device to scan the face. For this process, the device was always moved from the front center of the face to the sides in the same way in order to record both sides of the face. The data were then exported as a stereolithography file (.stl).

For the manual photogrammetry, a video recording of the face was made with a Nikon D5500 (Nikon Corporation, Tokyo, Japan) digital single-lens reflex camera (DSLR) with a suitable lens: the AF-P DX NIKKOR 18–55 mm (f/1:3.5–5.6G VR, Nikon Corporation). Starting from the forehead, the camera was moved clockwise around the face in order to capture all sides of the face from different perspectives. The file was then saved in QuickTime File Format (.mov). The hardware used was available for EUR 899 and the software was open source.

### Post-processing and measurement

The.stl file from the smart device was imported into MeshLab (version 2020.03, Consiglio Nazionale delle Ricerche, Rome, Italy) for Windows. Next, artifacts were removed and the model was cut to the edges of the face. The file was then exported, again in.stl format.

**Fig. 5:**
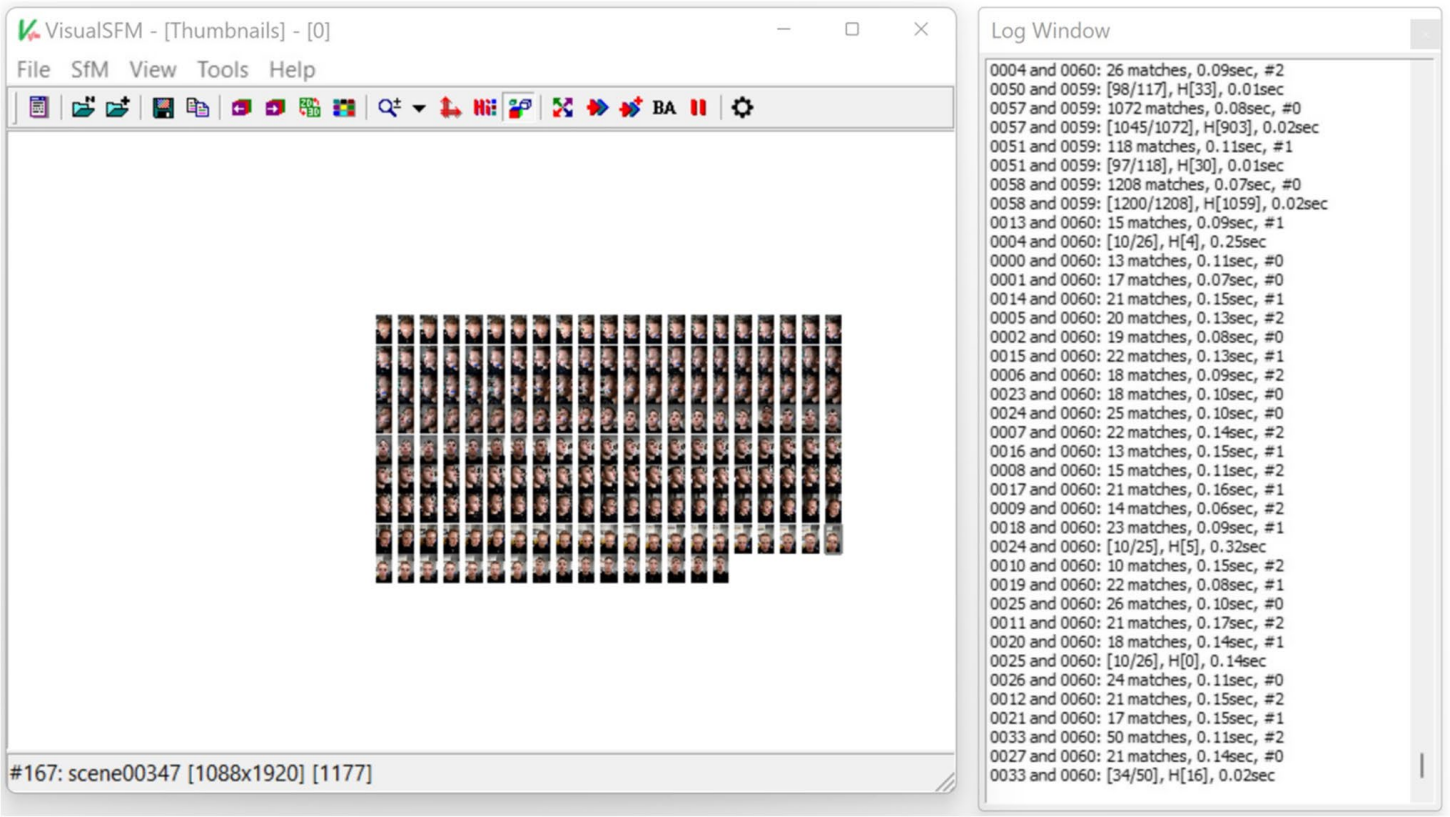
Import of the pictures taken with the video camera to VisualSFM software. Blurred pictures were previously removed

**Fig. 6:**
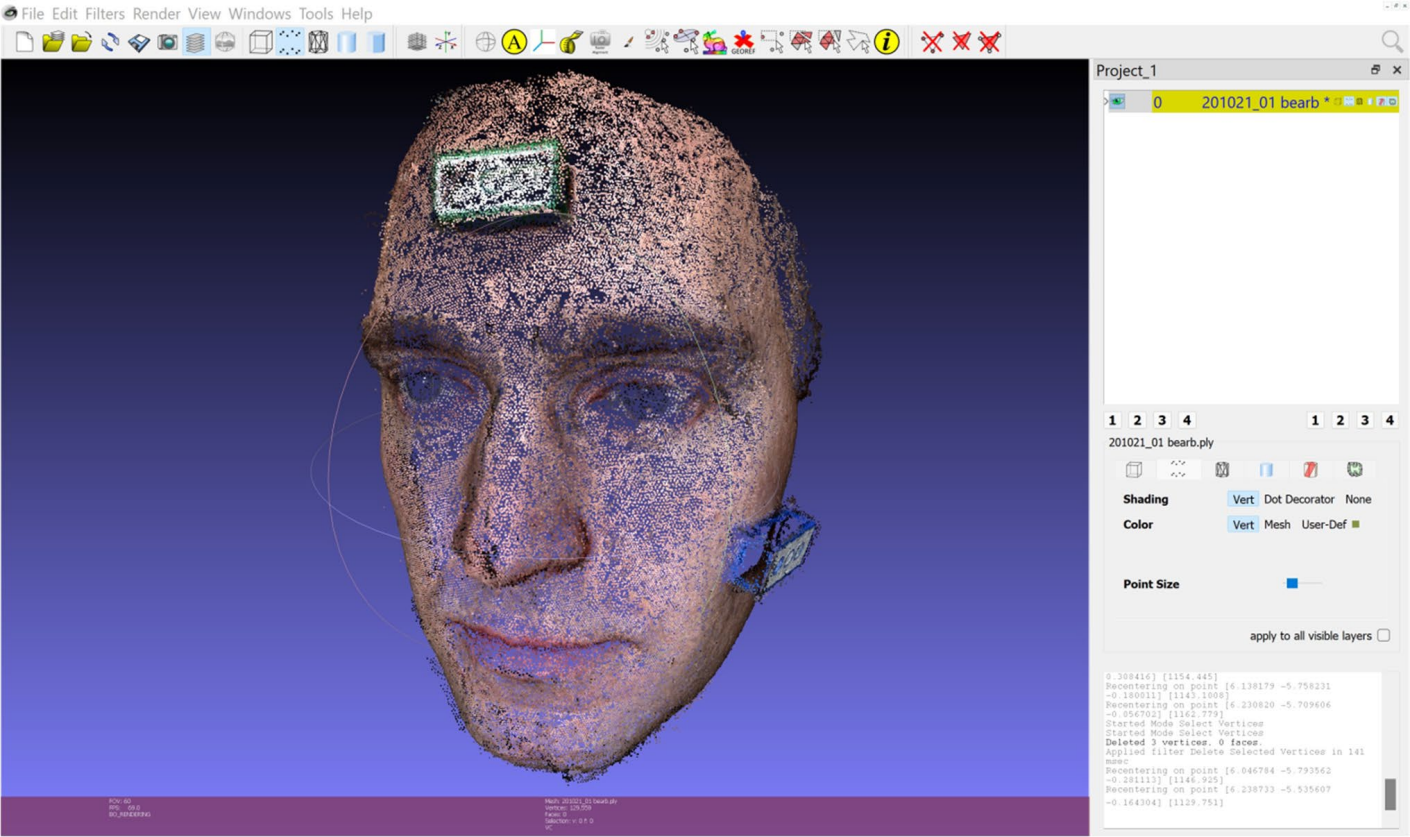
Reconstruction of the three-dimensional picture in Meshlab after removing of artifacts

Video files from the manual photogrammetry were imported into VLC Media Player (version 3.0.11 Vetinari, VideoLAN, Paris, France) for Windows. Using the scene filter function, single frames from the video were saved as image files in Joint Photographic Experts Group format (.jpg). Blurred photos were removed from the data set in order to only use suitable photos in the calculation of the 3D model. Next, a point cloud was calculated from the adjusted data set using a visual structure from motion system (SfM) GUI application: VisualSFM (version 0.5.26, developer: Changchang Wu) for Windows (Fig. 5). This was saved as a Polygon File Format (.ply) and imported into MeshLab (Fig. 6). As for the models created with the smart device, artifacts were removed and the face was cut to the edges. The normals of the point cloud were calculated and a surface reconstruction was carried out. This result was also exported in.stl format.

GOM Inspect 2019 (Hotfix 8, GOM GmbH, Braunschweig, Germany) for Windows was used for the measurement of the parameters in both data sets. Because the.stl data sets did not have a reference for the adequate measuring of distances, the models were scaled based on the length of the brick attached to the forehead (31.8 mm). For this purpose, a fitting plane was constructed on the frontal side of the test item with output of the dimensions. Then, all further fitting planes were constructed using selected points on the outer edges of both test items. These planes were calculated using the Gauss best-fit method based on three sigma of the selected points in order to correct outliers. Thus, there were five fitting levels per test item. Next, the remaining dimensions of the two frontal fitting planes of both test items—except the scaled length—were documented and all possible angles between the constructed fitting planes were measured. This gave eight angles per test item. Furthermore, the abovementioned distances based on anatomical landmarks were measured as a two-point distance following manual selection of the respective points. The scans for the smart device are shown in Fig. 3 and those for the photogrammetry technique are presented in Fig. 4.

### Statistical analysis

Statistical analysis was performed using IBM SPSS Statistics (version 25, IBM Corporation, New York, USA). The dependent *t*-test was applied to the metric values and means and standard deviation were calculated for all values. Normal distribution was calculated and confirmed via evaluation of a Q–Q plot.

## Results

Deviation of the anthropometric distances was calculated for all previously defined data sets, leading to a total of eight values. On average, they deviated 2.17 mm from the manual measurements, and photogrammetry demonstrated higher accuracy than smart device scanning (deviation 1.34 vs. 3.01 mm). Nonetheless, all of the eight deviations were statistically significant.

For the measurement of the length and width of the bricks, two out of three smart device measurement values differed significantly from the defined values. Similarly, two out of three manual photogrammetry values also differed significantly from the defined values (Tables 1 and 2).

**Table 1:** Comparison of the face scan using a smart device and manual measurements, and defined lengths and angles

	Smart device (SD)	Manually/defined (SD)	*p* values
Anthropometric values			
Medial canthi	34.12 (± 3.16)	31.00 (± 3.47)	0.004
Lateral canthi	98.40 (± 7.15)	94.39 (± 6.67)	0.000
Nasal alae	36.95 (± 3.86)	35.72 (± 3.62)	0.000
Mouth	55.74 (± 4.61)	52.03 (± 3.91)	0.000
Dimensions of the gaming brick			
Width forehead	15.20 (± 0.96)	15.80	0.000
Length cheek	32.41 (± 1.52)	31.80	0.007
Width cheek	16.06 (± 1.10)	15.80	0.105
Angles of the gaming brick			
Left to top plane forehead	98.12 (± 5.93)	90	0.000
Right to top plane forehead	97.60 (± 4.45)	90	0.000
Right to bottom plane forehead	90.80 (± 3.49)	90	0.111
Left to bottom plane forehead	94.20 (± 4.98)	90	0.000
Left to front plane forehead	105.69 (± 8.56)	90	0.000
Top to front plane forehead	118.56 (± 10.71)	90	0.000
Right to top plane forehead	98.94 (± 6.86)	90	0.000
Bottom to front plane forehead	107.73 (± 6.36)	90	0.000
Left to top plane cheek	90.09 (± 3.26)	90	0.842
Right to top plane cheek	91.08 (± 3.81)	90	0.050
Right to bottom plane cheek	94.01 (± 4.72)	90	0.000
Left to bottom plane cheek	91.17 (± 3.65)	90	0.028
Left to front plane cheek	97.74 (± 6.31)	90	0.000
Top to front plane cheek	98.81 (± 6.90)	90	0.000
Right to top plane cheek	95.87 (± 8.95)	90	0.000
Bottom to front plane cheek	104.78 (± 7.45)	90	0.000

**Table 2:** Comparison of the face scan using manual photogrammetry and manual measurements, and defined lengths and angles

	Photogrammetry (SD)	Manually/defined (SD)	*p* values
Anthropometric values			
Medial canthi	32.27 (± 2.83)	31.00 (± 3.47)	0.000
Lateral canthi	92.06 (± 6.41)	94.39 (± 6.67)	0.000
Nasal alae	34.28 (± 3.30)	35.72 (± 3.62)	0.000
Mouth	52.35 (± 4.34)	52.03 (± 3.91)	0.000
Dimensions of the gaming brick			
Width forehead	16.52 (± 1.01)	15.80	0.000
Length cheek	32.09 (± 1.02)	31.80	0.050
Width cheek	17.19 (± 1.12)	15.80	0.000
Angles of the gaming brick			
Left to top plane forehead	89.45 (± 3.14)	90	0.224
Right to top plane forehead	90.72 (± 3.70)	90	0.173
Right to bottom plane forehead	89.43 (± 3.29)	90	0.229
Left to bottom plane forehead	90.76 (± 3.17)	90	0.097
Left to front plane forehead	91.61 (± 4.81)	90	0.022
Top to front plane forehead	92.19 (± 5.06)	90	0.004
Right to top plane forehead	89.29 (± 4.15)	90	0.233
Bottom to front plane forehead	93.72 (± 5.49)	90	0.000
Left to top plane cheek	90.68 (± 3.32)	90	0.156
Right to top plane cheek	91.83 (± 3.41)	90	0.000
Right to bottom plane cheek	89.21 (± 5.47)	90	0.313
Left to bottom plane cheek	90.48 (± 4.33)	90	0.434
Left to front plane cheek	90.25 (± 5.83)	90	0.763
Top to front plane cheek	90.93 (± 3.62)	90	0.076
Right to top plane cheek	93.11 (± 8.09)	90	0.009
Bottom to front plane cheek	100.30 (± 9.76)	90	0.000

To assess the geometric reliability, a total of eight angles on each brick and for each face scanner were measured, leading to a total of 32 values. The majority of them (19 of 32) demonstrated a significant difference to the 90° angle of the bricks. Thirteen of these were from smart device scanning and six were from manual photogrammetry. The average deviation was 6.5°. However, manual photogrammetry (mean deviation of 2.8°) demonstrated higher accuracy in comparison with smart device capturing (10.1° deviation) (Tables 1 and 2). The measured angles appeared to be too large, especially for the smart device.

For manual photogrammetry, 151.18 (± 30.62) pictures were taken for each. stl data set.

## Discussion

The use of face scanners to obtain 3D facial images has become increasingly popular over recent decades, especially in the field of maxillofacial and aesthetic surgery. There are several ways in which to utilize face scan images, such as in the evaluation of volumetric changes after surgical interventions and the preoperative or postoperative evaluation of surgery. However, several different models exist with varying quality in terms of the data sets produced and the consequent reconstructed 3D faces.

Camison et al. calculated the distances between several marked points on the face and reported 136 distances in total. The deviation was, on average, 0.84 mm [2]. Other authors have used heatmaps to determine a mean absolute difference, resulting in an accuracy of between 0.32 and 0.71 mm. The same technique was also used for an iPad sensor accessory, resulting in an accuracy of 1.33 mm [3, 6]. In the present study, we achieved an average deviation of 1.34 mm with photometry and 3.01 mm using a smart device. Since the distance between anthropometric points was measured, the results are comparable to those of Camison et al.; however, they are technically not comparable to the measurement of a heatmap. Using Lego bricks, Modabber et al. determined a mean deviation of 0.42° to 35.41° from the 90° angles for a professional face scanner [4]. Surprisingly, both techniques assessed in our study achieved better results for the measurement of the angles than the professional face scanner did. The reason for this may not be related to the device used to capture the 3D data, but rather to the software of the processors as they tend to smoothen edges. This effect was also seen in the cohort of patients measured with the smart device: the angles were larger than in reality, which can likely be attributed to the smoothening effect.

In our study, manual photogrammetry with a regular photo-camera resulted in more detailed and more accurate data than those produced using the smart device. However, the major disadvantage to manual photogrammetry is the data acquisition. There were, on average, 151.18 ± 30.26 photos required, all of which needed to be fused. Therefore, manual photogrammetry does not appear to be a suitable solution for routine imaging. Nonetheless, in distinct cases—such as research settings—it may represent a suitable alternative.

By contrast, scanning with a smart device was more user friendly and intuitive but resulted in less accurate reconstructions of the face. Over the long term, smart devices are likely to improve further in terms of both camera capabilities and processing software. It appears likely that these devices will be able to deliver 3D data sets comparable to professional cameras in the near future. Thus, regular analysis of every new generation of camera-enabled smart devices should be performed.

In daily practice, one use of 3D face scans is the production of individual protective masks for athletes. Cazon et al. compared two scanners and found a deviation of the mask from the scanned surface of between − 2.0 and 2.7 mm—with an average of 0.18 and 0.15 mm—for the two scanning devices [9]. In a case series report by Steiner et al., a face mask produced by conventional plaster impression demonstrated an average deviation of 1.57 mm, with a maximum deviation of 5.62 mm. It was then compared with a production based on a 3D scan. The 3D scan-based mask demonstrated an average deviation of 0.99 mm and a maximum deviation of 6.18 mm. They concluded that differences of a few millimeters do not seem to reduce the comfort or protective effect of these masks [10]. In our study, the average deviation of the anthropometric measurements ranged from 1.34 to 3.01 mm. Thus, the investigated techniques may also be appropriate for the production of protective masks.

Amornvit et al. also described the use of an iPhone for face scanning. They used the Bellus3D app for data acquisition [11]. Additional potential software programs include Trnio, Capture, and Scandy Pro. However, in the evaluation phase—prior to the conduction of the study—all of the obtained images obtained with the Bellus3D app were either not precise enough or resulted in problems with data export. Therefore, the authors chose the Heges app as the most suitable for use on an iOS device. Android-driven or other devices were not considered in the study but certainly provide reliable alternatives.

Finally, it is important to mention that pictures from photogrammetry do not appear as smooth as the 3D pictures from smart devices, as previously described. This is important to note for daily practice: if the scan is required for patient education, a smoothened surface is desirable and minor discrepancies in length or angles may not constitute a problem. By contrast, for the purposes of research, the more detailed the face scan and the less deviation seen, the better. Thus, photogrammetry seems to be the favorable option in these cases.

In summary, we believe that smart devices appear to be adequate for patient education despite errors of up to 3 mm. Easier handling and the smoother surfaces of 3D models make them more likely to be used in this context. In research settings, this may not be adequate and alternatives should be considered, including photogrammetry.

Further studies may seek to analyze the ease of use of these techniques for both professionals and laypeople. Particularly in cases where face scans may be delegated to nursing staff, such studies may provide important insights and thus should be considered in the future.

## Conclusion

Manual photogrammetry with a regular photo-camera demonstrated higher accuracy then scanning with a smart device but with much greater complexity involved during processing to obtain 3D facial images. Thus, this technique may be more suitable in some cases—especially in research settings. Smart device scanning is more intuitive and could be preferable in patient education contexts. This technology is undergoing massive technical development and clinical reevaluation in the near future is likely to be of interest.
